# Compact metasurface track-side PIS antenna-based occupational electromagnetic exposure research of inspectors in the metro tunnel

**DOI:** 10.3389/fpubh.2026.1893518

**Published:** 2026-09-03

**Authors:** Ming-Fei Luo, Jin-Jing Xu, Wen-Ying Zhou, Xin-Hong Li, Mai Lu

**Affiliations:** 1 Key Laboratory of Opto-Electronic Technology and Intelligent Control of Ministry of Education, Lanzhou Jiaotong University, Lanzhou, China; 2 School of Automation and Electrical Engineering, Lanzhou Jiaotong University, Lanzhou, China; 3 State Key Laboratory of Millimeter Waves, Nanjing, China

**Keywords:** compact metasurface track-side PIS antenna, closed electromagnetic environment, occupational radio-frequency (RF) exposure, SAR, track-side inspectors

## Abstract

**Introduction:**

With the advancement of Smart Urban Rail construction, the electromagnetic environment has become increasingly complex inside metro tunnels. Track-side inspectors may experience occupational radio-frequency (RF) exposure when conducting professional inspections in such a closed electromagnetic environment.

**Methods:**

In order to analyze the occupational electromagnetic exposure dose absorbed by the track-side inspectors,and explore a method to reduce the influence of RF electromagnetic radiation. We propose a compact metasurface track-side passenger information system (PIS) antenna at first, and verify its good radiation performance. Then we compare the electromagnetic exposure metrics in the track-side inspectors human model when they are exposed to the conventional track-side PIS antenna and the proposed track-side PIS antenna at 5.8 GHz.

**Results:**

The simulation results show that, the peak local specific absorption rate (SAR) is reduced by at least 39.83% in the trunk, skull, brain, heart, liver, and testes after the antenna is miniaturized by 53%.

**Discussion:**

The findings indicate that the proposed track-side PIS antenna can reduce the external radiation energy, thereby reducing its impact on the surrounding environment. This research provides a theoretical analysis for occupational RF exposure safety of track-side inspectors and offers a dosimetry reference for antenna design in metro tunnel communication systems.

## 1 Introduction

With the rapid expansion of metro systems, the complexity of their communication assurance networks has markedly increased. As the backbone of modern urban transportation, the safe and efficient operation of metro systems relies on the coordination between dedicated wireless networks (such as Communication-Based Train Control - CBTC, PIS) and public communication networks (such as 5G cellular systems). Dedicated communication systems manage train dispatching, emergency communication, and passenger information, while public communication systems primarily serve passengers’ daily communication needs. The growing demand for both types of communication systems has led to an increasing number of wireless radiation sources being put into operation,which intensifies the complexity of the electromagnetic environment inside metro tunnels (1). Unlike open environments, metro tunnels are enclosed spaces where electromagnetic waves undergo multiple reflections, diffractions, and modal couplings, resulting in intricate field distributions and pronounced multipath effects. These characteristics pose challenges not only to communication quality (2) and electromagnetic compatibility (3) but also to occupational exposure risk. Metro track-side inspectors are responsible for conducting professional inspections of the environment inside the tunnels every night after the metro trains have stopped running. Due to repeated long exposure to such a composite wireless communication environment, track-side inspectors are considered a potentially under-studied occupational group. Previous studies have investigated the thermal and non-thermal biological effects (4) associated with radio-frequency (RF) electromagnetic fields under various exposure conditions. For example, Stam (5) reviewed occupational exposure studies and found that in some scenarios, exposure levels might exceed safety limits, emphasizing the need to monitor emerging technologies such as 5G and wireless power transfer. Vila etal. (6) conducted a multinational case–control study of nearly 10,000 participants and reported no clear association between occupational exposure and brain tumor risk, although a non-significant increase was observed among recently exposed workers. Similarly, Bektas and Dasdag (7) reviewed the biological effects of RF electromagnetic radiation on male reproductive health, suggesting possible mechanisms involving oxidative stress, thermal load, and DNA damage. Against this backdrop, improving the electromagnetic environmental compatibility within tunnels and characterizing the electromagnetic exposure environment conditions experienced by inspectors is of practical significance.

Existing research on tunnel electromagnetic environments has largely focused on wireless signal coverage (8), propagation characteristics (9), and environmental modeling (10). For example, Zhao et al. (11) proposed a 5G metro tunnel communication system, achieving improved throughput and reduced penetration loss through dual connectivity and RAN sharing. Zhang et al. (12) conducted narrowband channel measurements, developing statistical models for various tunnel scenarios. However, these works mostly concentrate on improving communication performance and optimizing the overall electromagnetic environment, which highlights the need for further research on methods to enhance the electromagnetic compatibility of tunnels. Existing studies have shown that RF electromagnetic radiation can impact human health (13). Therefore, in metro tunnel environments, considerations of system performance and communication quality should be accompanied by attention to human exposure safety, particularly for occupational groups (14). Current studies on electromagnetic exposure safety in rail transit scenarios have mainly focused on: for example, Zhou et al. (15) analyzed the effects of different leaky coaxial cables on passenger SAR and temperature rise through simulation. Li and Lu (16) also examined station staff exposure under similar conditions and confirmed compliance with ICNIRP limits. However, most research has focused on passengers, with limited investigation into track-side inspectors. Therefore, a systematic assessment of their RF exposure environment and corresponding dosimetric analysis are necessary.

This paper initially presents a compact metasurface track-side PIS antenna. This antenna is capable of covering the PIS dedicated network frequency band (5.73 ~ 5.85 GHz) and concurrently supporting the public 5G n79 band (4.8 ~ 4.9 GHz). Subsequently, the radiation performance of the proposed track-side PIS antenna is compared with that of the conventional track-side PIS antenna, and their corresponding RF exposure dose are simulated under the same deployment configuration. The electric field strength, specific absorption rate (SAR), and temperature rise in the trunk, skull, brain, heart, liver, and testes of track-side inspectors are analyzed at 5.8 GHz. The comparative results indicate that, the electromagnetic dose absorbed by various body tissues of inspection personnel is reduced when track-side antenna is miniaturized. This study explores the far-field radiation effects of RF exposure sources miniaturization in subway tunnels on occupational populations.

## 2 Proposed track-side PIS antenna modeling and performance analysis

### 2.1 Geometric model comparison of track-side PIS antennas

**Figure 1:**
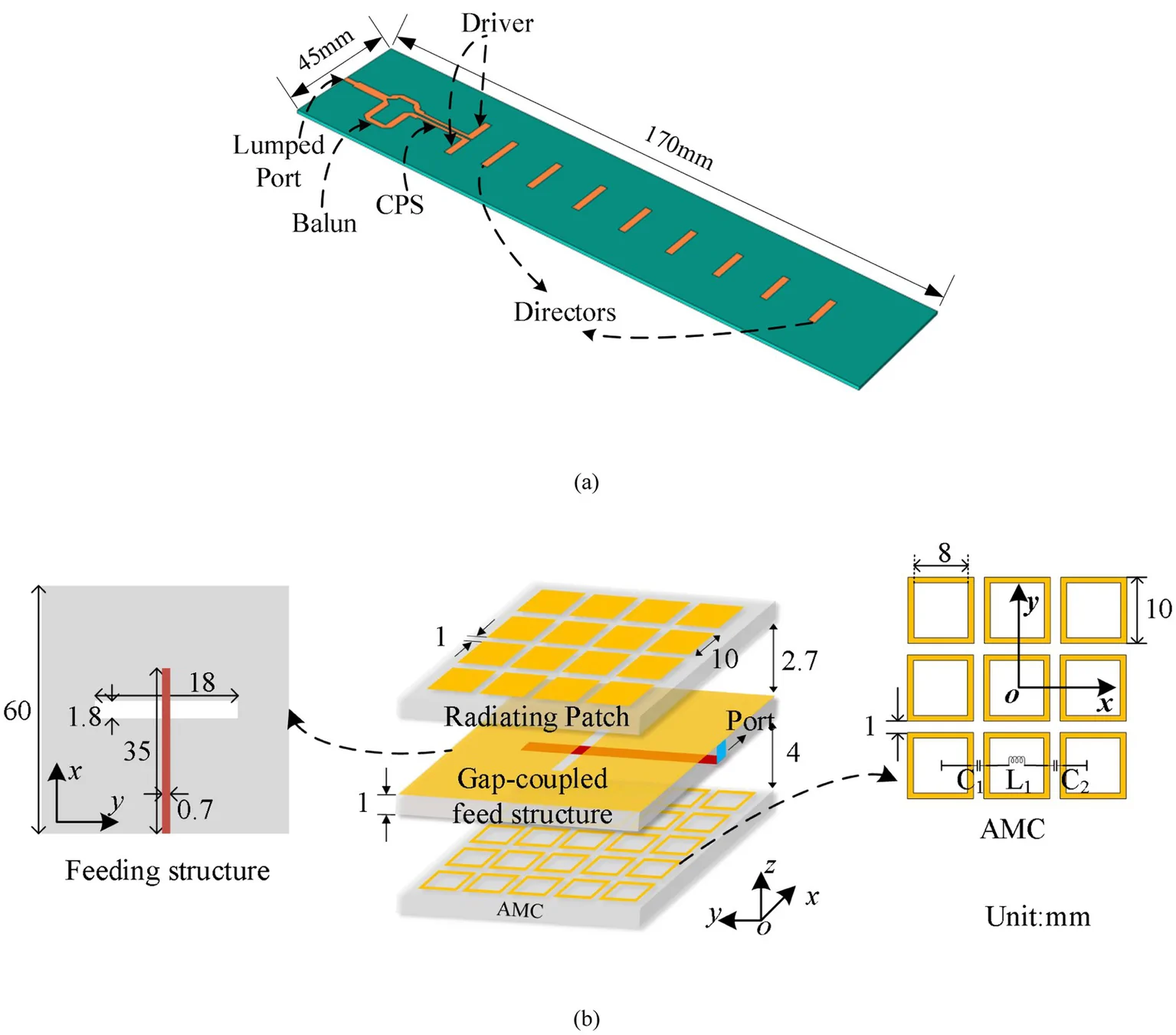
Geometry of the track-side PIS antenna. **(a)** Conventional track-side PIS antenna; **(b)** Proposed track-side PIS antenna.

Conventional track-side PIS antennas are typically Yagi-uda antenna, which consist of an active dipole, a passive reflector, and several passive directors arranged in parallel to achieve directional high-gain radiation at a fixed frequency. The conventional track-side PIS antenna operating at 5.8 GHz has dimensions of 45 mm × 170 mm, as illustrated in Figure 1a. The geometry of the proposed track-side PIS antenna designed in this paper is shown in Figure 1b, with a radiating area that is 53% smaller than that of the conventional track-side PIS antenna.

As shown in Figure 1b, the radiating area of the proposed track-side PIS antenna is only 60 mm × 60 mm. The antenna comprises three parallel dielectric layers: the top layer is the radiating patch, the middle layer is the aperture-coupled feed structure, and the bottom layer is an Artificial Magnetic Conductor (AMC). The dielectric substrate has a permittivity of 2.65, a loss tangent of 0.002, and a thickness of 1 mm each. The aperture-coupled feed structure, placed beneath this metasurface radiating unit, consists of a microstrip line and a narrow slot etched in the ground plane. The distance between the first and second dielectric layers is 2.7 mm, and between the second and third layers is 4 mm.

### 2.2 Performance analysis of the proposed track-side PIS antenna

**Figure 2:**
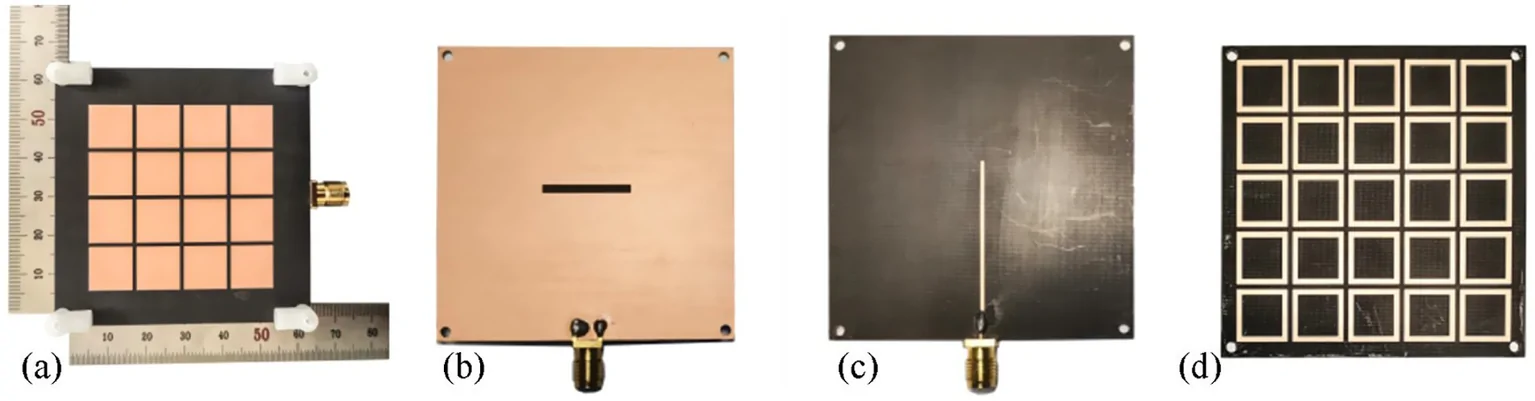
Prototype of the proposed track-side PIS antenna. **(a)** Top layer; **(b)** Middle layer front; **(c)** Middle layer back; **(d)** Bottom layer.

**Figure 3:**
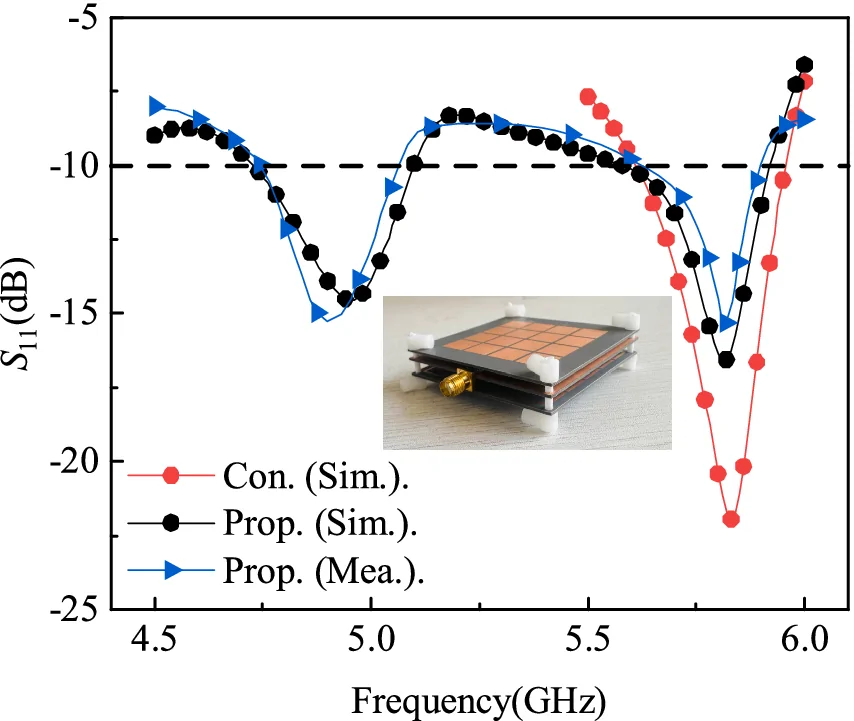
Comparison of measured and simulated *S*_11_ parameters.

To verify the performance of the proposed track-side PIS antenna, this study carried out a simulation comparison of S-parameters between the conventional track-side PIS antenna and the proposed track-side PIS antenna. A physical prototype of the proposed track-side antenna was fabricated (as shown in Figure 2), and its *S*_11_ parameters were measured by means of the Agilent Technologies E5071C vector network analyzer. The prototype was fabricated with three F4B dielectric boards connected by nylon rods, with overall dimensions of 60 × 60 × 8.7 mm^3^. Figure 3 presents the comparison of *S*_11_ simulation results between the conventional and proposed track-side PIS antennas, along with the comparison between the measured and simulated *S*_11_ results of the proposed track-side PIS antenna.

Figure 3 shows that, the conventional track-side PIS antenna has only one operating band of 5.55 GHz ~ 5.92 GHz, the proposed track-side PIS antenna exhibits excellent impedance matching characteristics in both the 4.9 GHz and 5.8 GHz frequency bands. The simulation results show operational frequency bands of 4.72 ~ 5.10 GHz and 5.57 ~ 5.92 GHz, achieving dual-band resonance. The measured bandwidths are 4.76 ~ 5.04 GHz and 5.64 ~ 5.88 GHz. Although slight frequency deviations are observed in the measured results compared to the simulations due to processing and soldering losses, the overall trend of *S*_11_ remains consistent, without affecting the operational bandwidth of the designed antenna, thereby verifying the reliability of the proposed track-side PIS antenna. Compared to the conventional track-side PIS antenna, it can not only serve the PIS system for dedicated network communication but also cover the civilian 5G n79 band, effectively supplementing the coverage of civilian 5G networks in metro tunnels.

**Figure 4:**
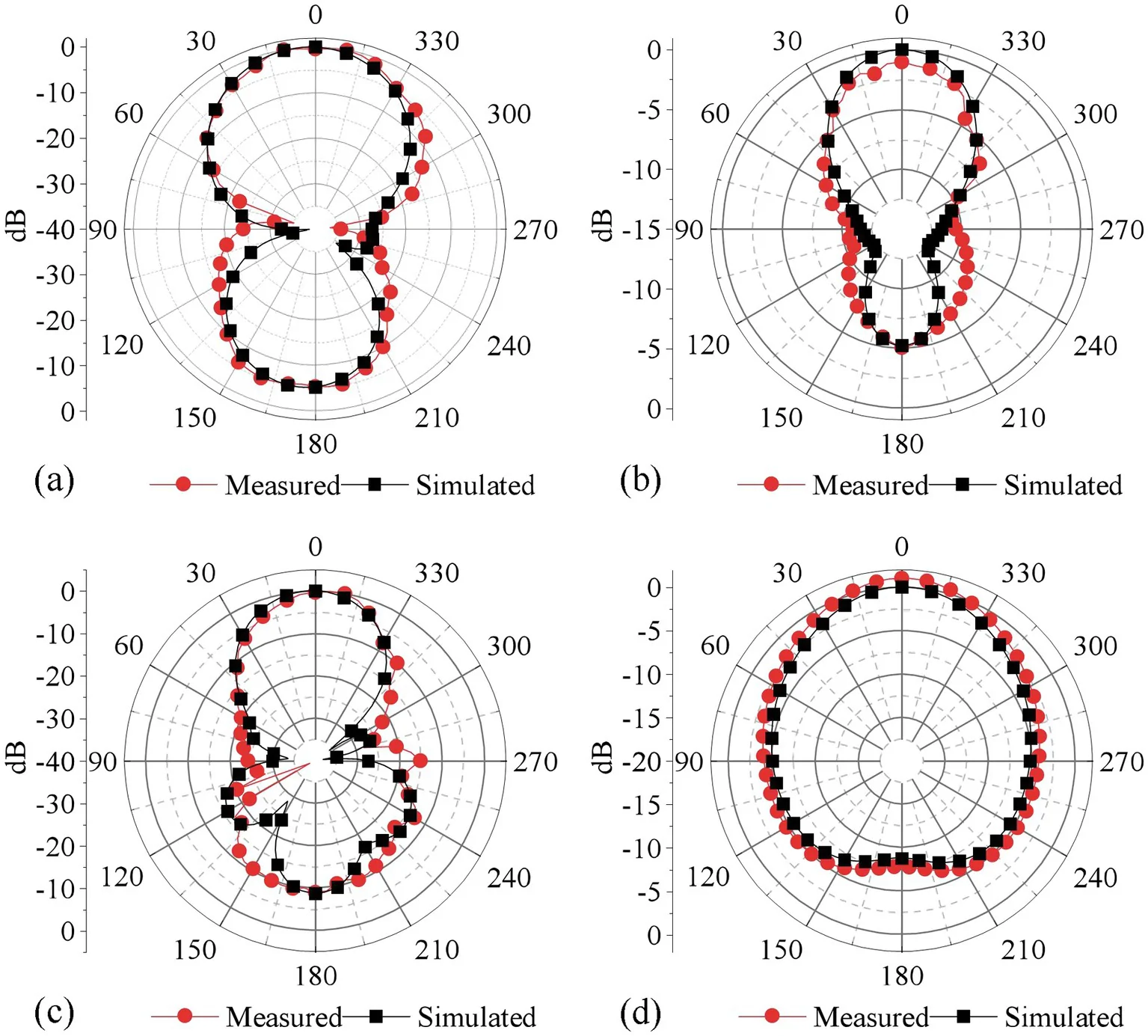
Normalized radiation patterns of the proposed track-side PIS antenna at 4.9 and 5.8 GHz. **(a)** E plane - 4.9 GHz; **(b)** H plane - 4.9 GHz; **(c)** E plane - 5.8 GHz; **(d)** H plane - 5.8 GHz.

The comparison of simulated and measured normalized radiation patterns at frequencies of 4.9 GHz and 5.8 GHz is shown in Figure 4.

Figures 4a,b show the normalized directional patterns of the E-plane and H-plane at 4.9 GHz, respectively. Figures 4c,d show the normalized directional patterns of the E-plane and H-plane at 5.8 GHz, respectively. From the Figure 4, it can be seen that the antenna has good directional performance in both the E-plane and H-plane, and the back lobe is larger compared with that of the conventional track-side PIS antenna, making it more suitable for wireless signal coverage in narrow metro tunnels. The simulation results are in good agreement with the measured results. This indicates that the gain remains essentially unchanged after reducing the antenna’s geometric size, meeting the communication requirements of the PIS system within the metro tunnel.

## 3 Electromagnetic exposure assessment for track-side inspectors in the tunnel

### 3.1 Exposure model

**Figure 5:**
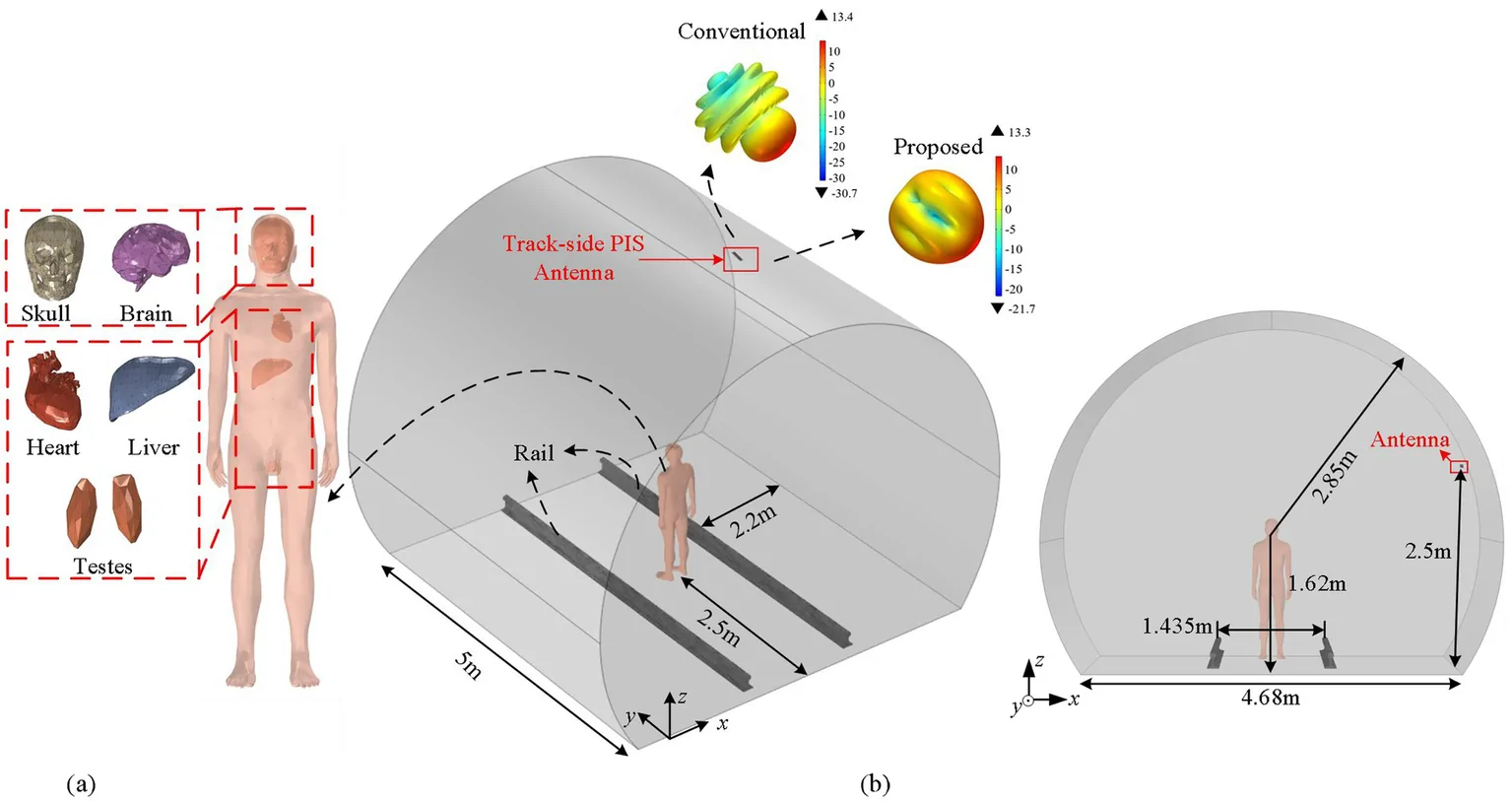
Human model and electromagnetic environment in the metro tunnel. **(a)** Human body model; **(b)** Electromagnetic environment surrounding the human body.

Given that track-side inspectors in metro tunnels are traditionally male-dominated (17), this study focuses on the occupational electromagnetic exposure of male inspectors. Within the metro tunnel, to guarantee operational safety, track-side inspectors generally conduct maintenance and inspection tasks during nightly non-operational hours while equipped with digital handheld devices (18). Considering this intricate electromagnetic environment, it is important to quantitatively evaluate the electromagnetic exposure levels for track-side inspectors. As indicated in Sciorio et al. (19), some studies have reported potential associations between RF electromagnetic radiation and male reproductive function under certain exposure conditions, with thermal effects and oxidative stress proposed as possible mechanisms. Moreover, Maluin et al. (20) further substantiates that RF electromagnetic radiation can reduce testosterone levels and potentially disrupt male reproductive hormones. Consequently, based on the internationally standardized adult male model (21), this paper constructs a human model incorporating reproductive organs (testes), as depicted in Figure 5a, to represent tunnel track-side inspectors for electromagnetic exposure safety assessment. Figure 5b shows that, at 5.8 GHz, the gain of the conventional track-side PIS antenna is 13.4 dBi, while that of the proposed track-side PIS antenna is 13.3 dBi.

**Figure 6:**
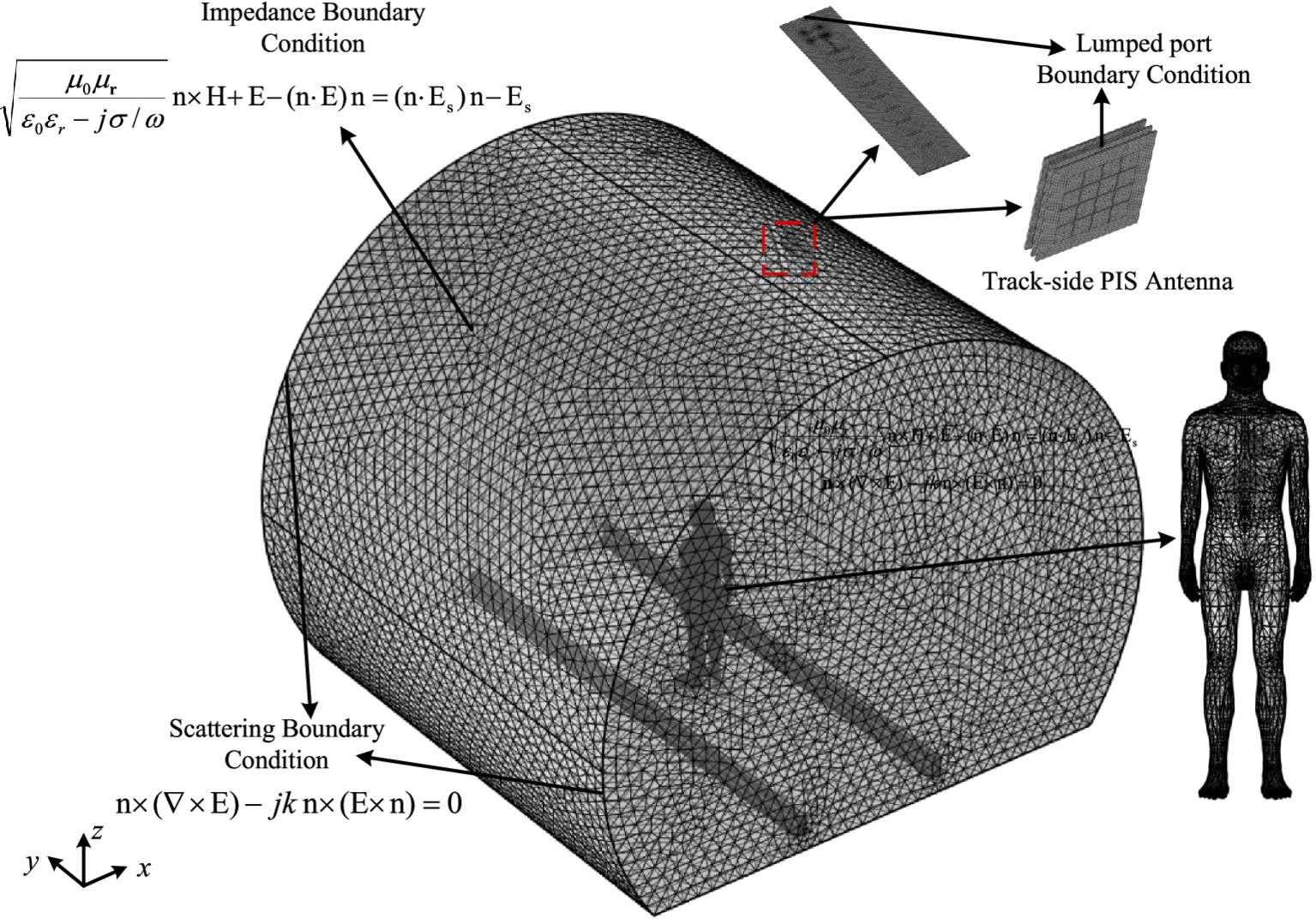
Mesh discretization of the 3D geometric model.

During the simulation, the antenna was excited using a lumped port. The tunnel boundaries were set as impedance boundary conditions, while the front and rear ends of the tunnel were set as scattering boundary conditions. The governing equations were solved under predefined initial and boundary conditions using COMSOL Multiphysics 6.2. The electromagnetic field calculations were performed using the Electromagnetic Waves, Frequency Domain interface from the RF Module, and the temperature response calculations were carried out using the Bioheat Transfer interface. The electromagnetic field and temperature field were coupled in a sequential manner, where the SAR distribution obtained from the frequency-domain electromagnetic simulation was imported as an external heat source into the bioheat transfer model. A direct solver was employed for the solution, with the relative tolerance set to 0.01. The model surfaces were discretized using triangular elements, and the three-dimensional computational domain was meshed with tetrahedral elements. Considering both computational accuracy and capability, local mesh refinement was applied to the antenna, regions with complex human anatomical structures, and fine geometric features where rapid electromagnetic field variations were anticipated. The final computational mesh consisted of 918,073 elements and approximately 5,900,690 degrees of freedom. The mesh model is shown in Figure 6.

The human model includes major tissues such as the trunk, skull, brain, heart, liver, and testes. The dielectric properties of the tissues in the human model at 5.8 GHz were calculated using the 4th-order Cole-Cole model proposed by Gabriel (1996) (22), as detailed in Table 1.

**Table 1:** The human tissue dielectric parameter at 5.8 GHz.

Tissue	5.8 GHz		*ρ*(kg/m^3^)
	$\varepsilon_{r}$	*σ*(S/m)	
Trunk	36.45	4.09	1,297
Skull	9.67	1.15	1990
Brain	38.31	4.42	1,038
Heart	48.95	5.86	1,050
Liver	38.13	4.64	1,151
Testes	52.21	5.95	1,050

Where $\varepsilon_{r}$ is the relative permittivity, σ is the conductivity, and ρ is the tissue density. The human model was positioned in the tunnel electromagnetic environment facing the main lobe radiation direction of the antenna, located at the center of the track, as shown in Figure 5b.

To study the temperature rise in different tissues of the human model, Table 2 provides the thermal parameters of the relevant tissues, where *k* is the thermal conductivity, *C* is the specific heat capacity, $\omega_{b}$ is the blood perfusion rate, and $Q_{\mathrm{met}}$ is the metabolic heat source. These thermal parameters were obtained from the IT’IS database (23).

**Table 2:** Thermal parameters of human tissues.

Tissue	*k*(W∙(m∙°C)^−1^)	*C*(J∙(kg∙°C) ^−1^)	$\omega_{b}$ (1/s)	$Q_{\mathrm{met}}$ (W/m^3^)
Trunk	0.5	3,500	0.0017	847
Skull	0.3	1,300	0.00017	298.5
Brain	0.5	3,650	0.00932	11,495
Heart	0.6	3,700	0.0171	24,000
Liver	0.52	3,600	0.01433	10,714
Testes	0.45	3,500	0.00333	3,399

### 3.2 Electric field strength distribution in tunnel and reliability verification

To clarify the electric field strength of tunnel environment, we use an R&S®FSH handheld spectrum analyzer to measure the signal power level of PIS dedicated network communication system at frequency of 5.8 GHz in metro tunnel.

The measurement method was consistent with Zhou and Jacksha (24). During the measurements, the measurement equipment was moved along the track direction, and the received power was recorded at different positions from 0 to 100 m away from the track-side antenna. An omnidirectional dipole receiving antenna with a gain of 5 dBi and a standard impedance of 50 Ω was employed and it was positioned at the center of the metro track, 1.5 m above the ground. For the 50 Ω measurement system, the measured received power was converted into electric field strength according to the antenna factor method, as shown in Equation (1):

E=K+L+P+107 (1)

Where *E* is the electric field strength ( dBμV/m) , *K* is the antenna factor (dB/m), *P* is the measured signal strength (dBm), and *L* is the connecting cable loss (dB). The antenna gain *G* is usually used to calculate the antenna coefficient *K*, as shown in Equation (2).

$$K=20\mathrm{lg}(\frac{9.76}{\lambda \sqrt{G}})$$ (2)

Where *λ* is the wavelength (m).

**Figure 7:**
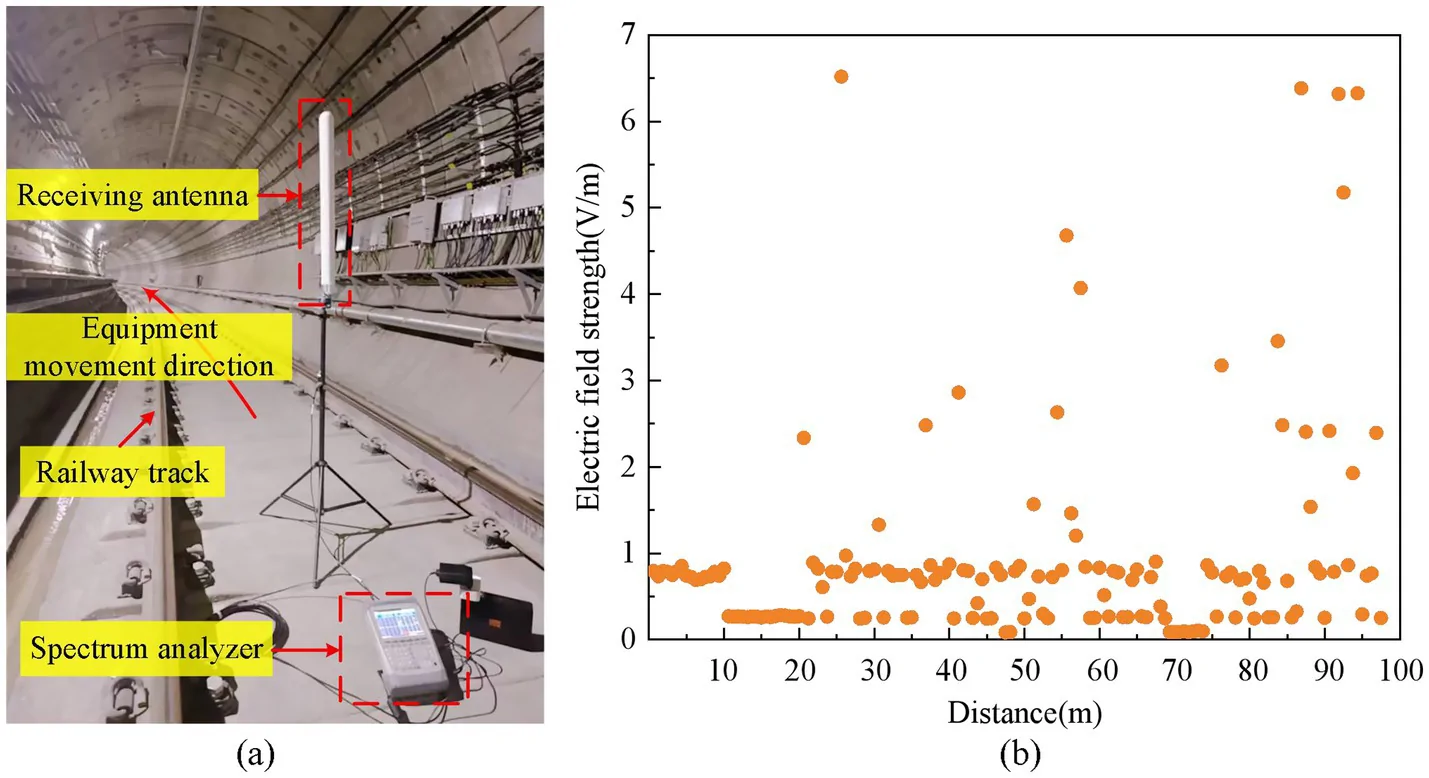
The test environment and the measured values. **(a)** Measurement setup in the metro tunnel; **(b)** Measured electric field strength versus distance.

The obtained electric field strength was further converted into V/m and compared with the simulation results. Based on the measured electric field strength, the input power of the numerical model was calibrated to 0.0006 W, so that the simulated electromagnetic field could represent the actual PIS electromagnetic environment in the metro tunnel. The test environment and the measured values are shown in Figure 7.

From Figure 7a, it can be seen that the measurement system consists of a measurement antenna, connecting cables, instruments, and a computer for data processing. A portable spectrum analyzer and an omnidirectional dipole antenna are used, with the antenna placed at the center of the metro tunnel rails, 1.5 m above the ground. By moving the measurement equipment parallel to the track direction, the power levels at distances ranging from 0 to 100 m away from the track-side antenna can be measured. As shown in Figure 7b, the received power measurements were converted into equivalent measured electric field strength values in the range of 0.08 to 6.51 V/m.

**Figure 8:**
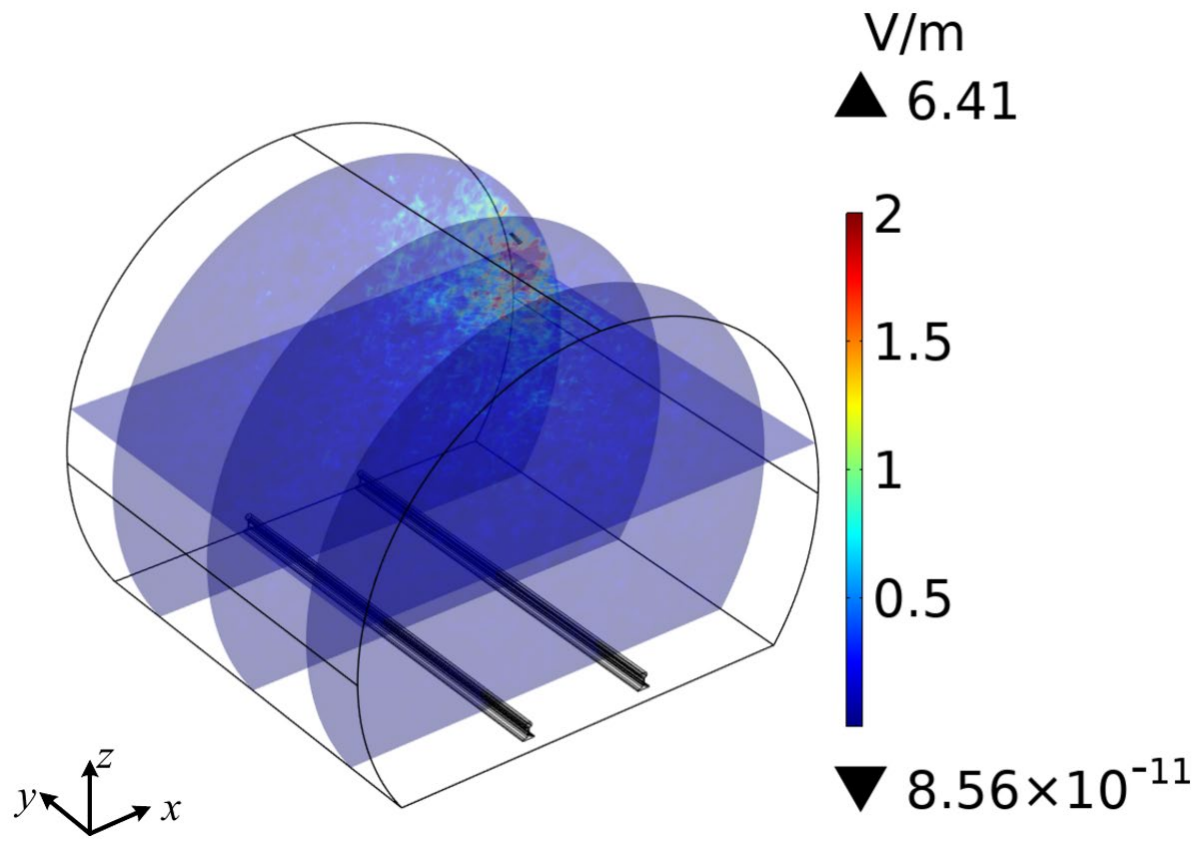
Electric field strength distribution in tunnel space.

The tunnel electromagnetic environment of a conventional track-side PIS antenna was simulated, and the electric field strength distribution inside the tunnel at the antenna operating frequency of 5.8 GHz was obtained, as shown in Figure 8.

From Figure 8, the maximum simulated electric field strength inside the tunnel reaches 6.41 V/m, while the electric field strength in the majority of the tunnel area remained below 0.80 V/m. Comparison with the measured results shows that the distribution range and peak variation characteristics of the electric field strength obtained using the equivalent model are in good agreement with the measurement data. These results indicate that the simulated model can effectively represent the electromagnetic environment characteristics inside the tunnel and provide a reliable foundation for further electromagnetic environment analysis.

### 3.3 Electric field strength distribution in track-side inspectors

**Figure 9:**
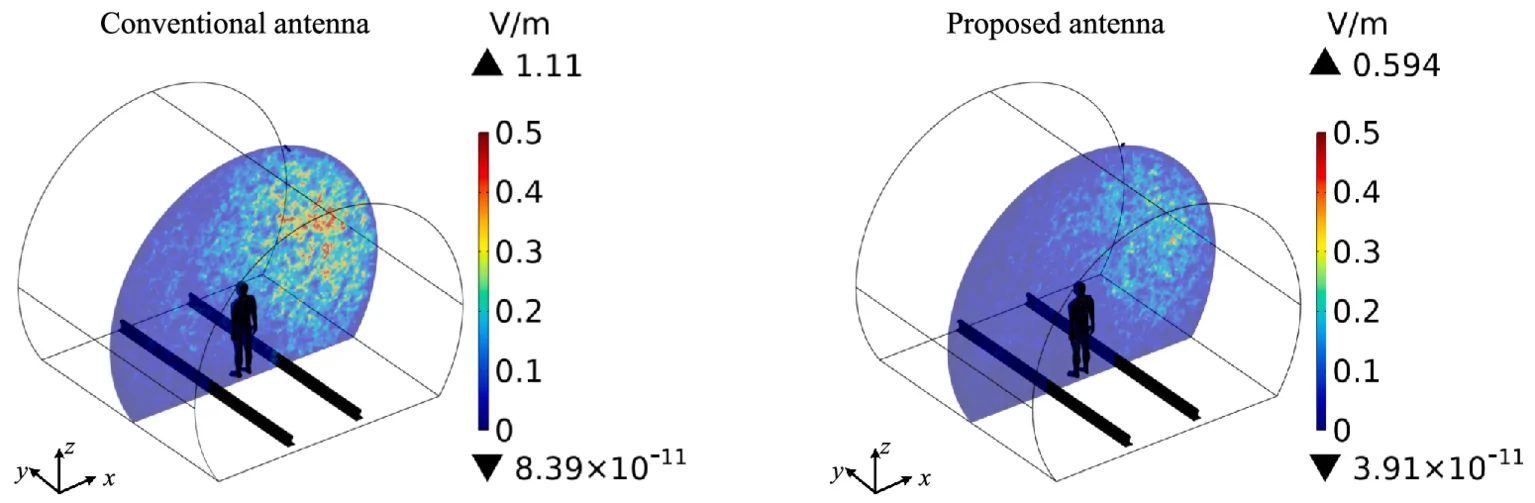
Electric field strength distribution in the tunnel cross-section occupied by the human body.

To further clarify the electromagnetic environment encountered by track-side inspectors at their actual working positions, Figure 9 illustrates the electric field strength distribution on the tunnel cross-section (*xoz* plane) where the human model is located.

As depicted in Figure 9, it is evident that the electric field strength within this cross-section is predominantly concentrated in the main lobe radiation direction of the antenna. Furthermore, when compared to the conventional track-side PIS antenna, the peak electric field strength in the tunnel cross-section at the position of the human body is decreased by 46.49% with the proposed track-side PIS antenna. This implies that the proposed track-side PIS antenna can effectively reduce the electric field strength in the area occupied by the human body.

**Figure 10:**
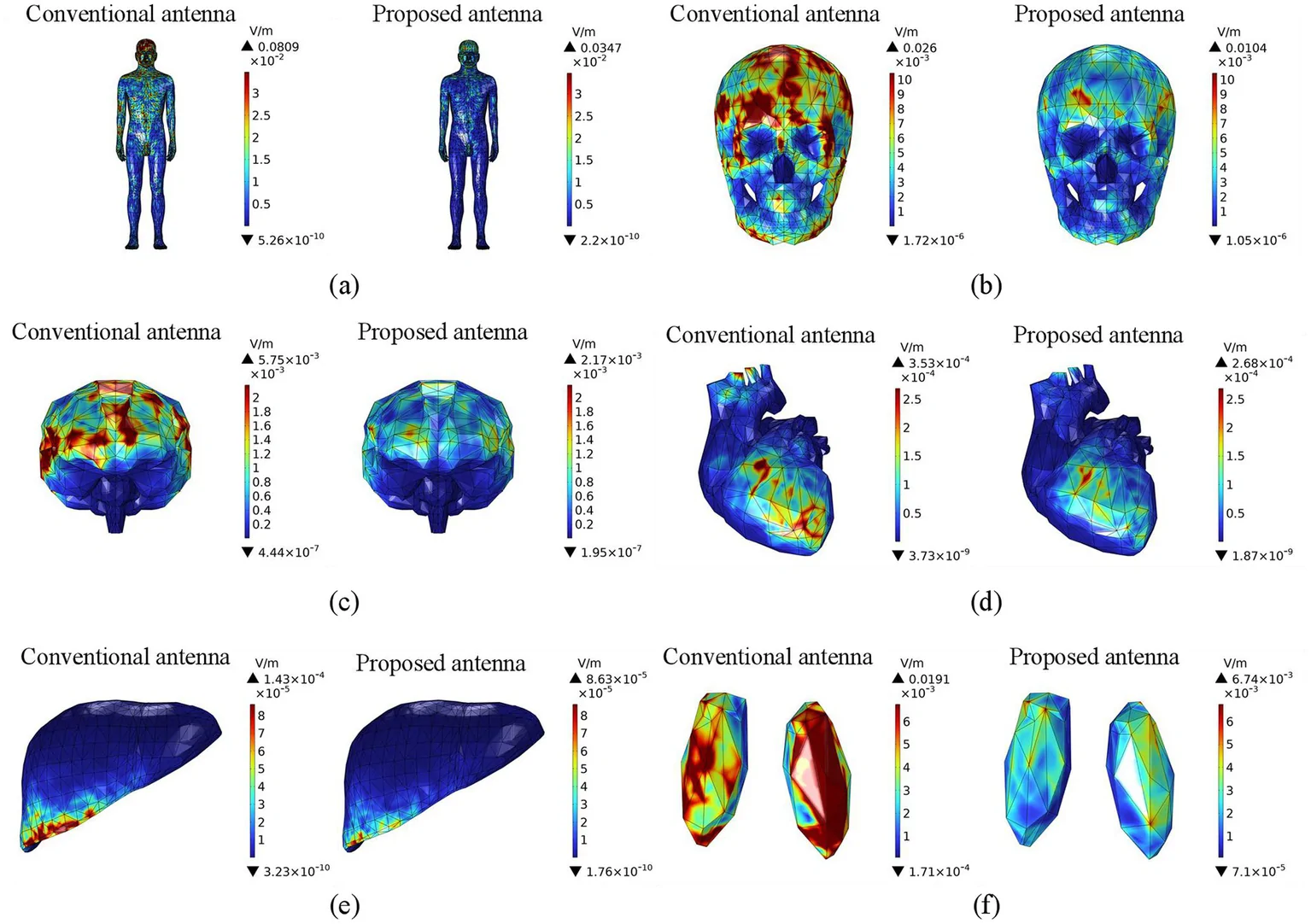
Comparison of electric field strength distribution in different human tissues. **(a)** Trunk; **(b)** Skull; **(c)** Brain; **(d)** Heart; **(e)** Liver; **(f)** Testes.

Upon clarifying the external field environment surrounding the human body, the distributions of electric field strength in various tissues under the irradiation of both conventional and proposed track-side PIS antennas at 5.8 GHz are simulated, as depicted in Figure 10. It shows the electric field strength distribution in the trunk, skull, brain, heart, liver, and testes at 5.8 GHz. For a quantitative comparison, the peak and spatial-average electric field strengths of different tissues are extracted from the simulation results and listed in Table 3.

**Table 3:** Comparison of electric field strength in different human tissues.

Tissue	Peak electric field strength (V/m)			Spatial-average electric field strength (V/m)		
	Conventional	Proposed	Reduction (%)	Conventional	Proposed	Reduction (%)
Trunk	0.0809	0.0347	57.11%	1.16 × 10^−3^	6.04 × 10^−4^	47.90%
Skull	0.026	0.0104	60.00%	1.71 × 10^−3^	8.71 × 10^−4^	49.10%
Brain	5.75 × 10^−3^	2.17 × 10^−3^	62.26%	1.82 × 10^−4^	8.92 × 10^−5^	51.00%
Heart	3.53 × 10^−4^	2.68 × 10^−4^	24.08%	1.01 × 10^−5^	6.69 × 10^−6^	33.80%
Liver	1.43 × 10^−4^	8.63 × 10^−5^	39.65%	1.41 × 10^−6^	7.73 × 10^−7^	45.20%
Testes	0.0191	6.74 × 10^−3^	64.71%	2.46 × 10^−3^	1.05 × 10^−3^	57.30%

Figure 10 demonstrates that under the irradiation of the proposed track-side PIS antenna, the electric field strength distribution varies notably among different human tissues. The maximum electric field strength in the trunk is mainly concentrated in the head, chest, and abdomen. In the skull, it is concentrated in the frontal bone, parietal bone, and mandible. The brain presents a relatively uniform distribution. The heart shows concentration in the left and right ventricles. The liver shows concentration in the right lobe. The testes, being superficially located and having a high water content, exhibit relatively higher electric field strength compared with other deeper-seated tissues.

In comparison with the conventional track-side PIS antenna, the proposed track-side PIS antenna substantially diminishes the electric field strength in various human tissues while sustaining good communication performance. When the antenna operates at frequency of 5.8 GHz, the peak electric field strength in the trunk experiences a reduction of 57.11%, in the skull by 60.00%, in the brain by 62.26%, in the heart by 24.08%, in the liver by 39.65%, and in the testes by 64.71%. This suggests that the metasurface track-side PIS antenna contributes to a decrease in the occupational electromagnetic exposure levels of track-side inspectors.

### 3.4 SAR distribution in human body

SAR is defined as the rate of electromagnetic energy absorption per unit mass of biological tissue and is calculated, as shown in Equation (3):

$$\mathrm{SAR}=\frac{\sigma {|E|}^{2}}{2p}$$ (3)

Where *σ* is the electrical conductivity of the tissue, *ρ* is the mass density of the tissue, and *|**E**|* is the magnitude of the electric field strength inside the tissue.

**Figure 11:**
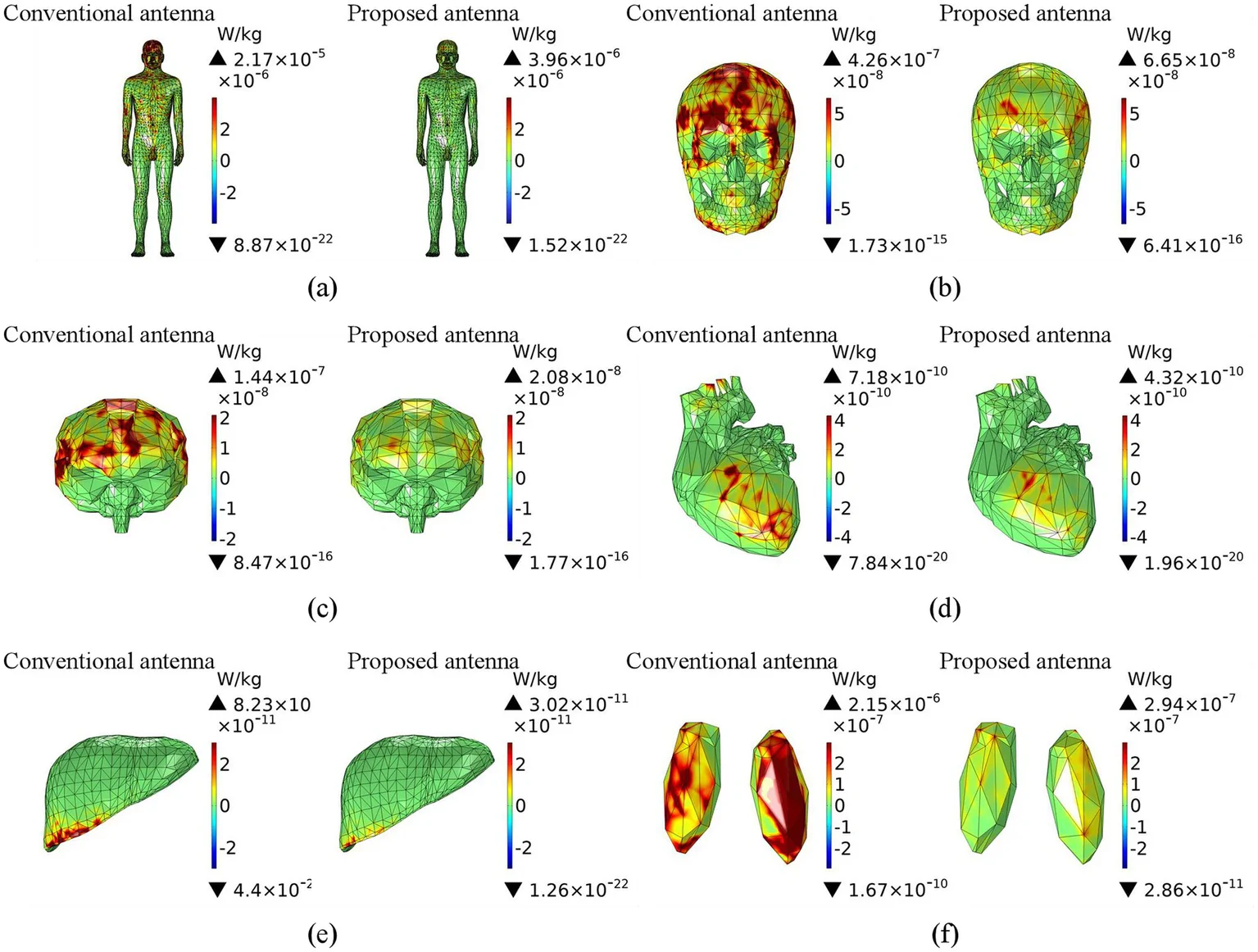
Comparison of SAR distribution in different human tissues. **(a)** Trunk; **(b)** Skull; **(c)** Brain; **(d)** Heart; **(e)** Liver; **(f)** Testes.

Based on the Finite Element Method, the SAR distributions in different human tissues under electromagnetic radiation from conventional and proposed track-side PIS antennas were simulated, as shown in Figure 11. The figure shows the SAR distribution in the trunk, skull, brain, heart, liver, and testes at 5.8 GHz. In addition, Table 4 summarizes the peak local SAR and the peak spatial-average SAR_10g_ values for different human tissues.

**Table 4:** Comparison of SAR values in different human tissues.

Tissue	Peak local SAR (W/kg)			Peak spatial-average SAR_10g_ (W/kg)		
	Conventional	Proposed	Reduction (%)	Conventional	Proposed	Reduction (%)
Trunk	2.17 × 10^−5^	3.96 × 10^−6^	81.75%	2.65 × 10^−6^	1.41 × 10^−6^	46.79%
Skull	4.26 × 10^−7^	6.65 × 10^−8^	84.39%	2.18 × 10^−7^	6.36 × 10^−8^	70.83%
Brain	1.44 × 10^−7^	2.08 × 10^−8^	85.56%	9.09 × 10^−9^	3.65 × 10^−9^	59.85%
Heart	7.18 × 10^−10^	4.32 × 10^−10^	39.83%	7.05 × 10^−10^	2.04 × 10^−10^	71.06%
Liver	8.23 × 10^−11^	3.02 × 10^−11^	63.30%	8.07 × 10^−11^	2.4 × 10^−11^	70.26%
Testes	2.15 × 10^−6^	2.94 × 10^−7^	86.33%	1.4 × 10^−7^	4.77 × 10^−8^	65.93%

Figure 11 illustrates that under the irradiation of the proposed track-side PIS antenna, the maximum SAR in the human trunk is predominantly distributed in the chest, abdomen, and head. Variations in the conductivity and permittivity of tissues such as bone and muscle result in non-uniform attenuation and distribution of the electric field strength as it traverses these tissues. When the field penetrates the trunk, the substantial absorption and scattering by muscle and adipose tissues reduces the field strength before it reaches the brain. As a result, the SAR values in the skull, brain, heart, and liver are considerably lower than those in the trunk. Among the non-trunk tissues, the testes exhibit the highest peak local SAR. Specifically, the peak local SAR of the testes is more than 5.05 times greater than the corresponding peak local SAR values of the skull, brain, heart, and liver. This difference may be attributed to the relatively high water content and dielectric properties of testicular tissue, which contribute to increased SAR values under electromagnetic exposure.

**Figure 12:**
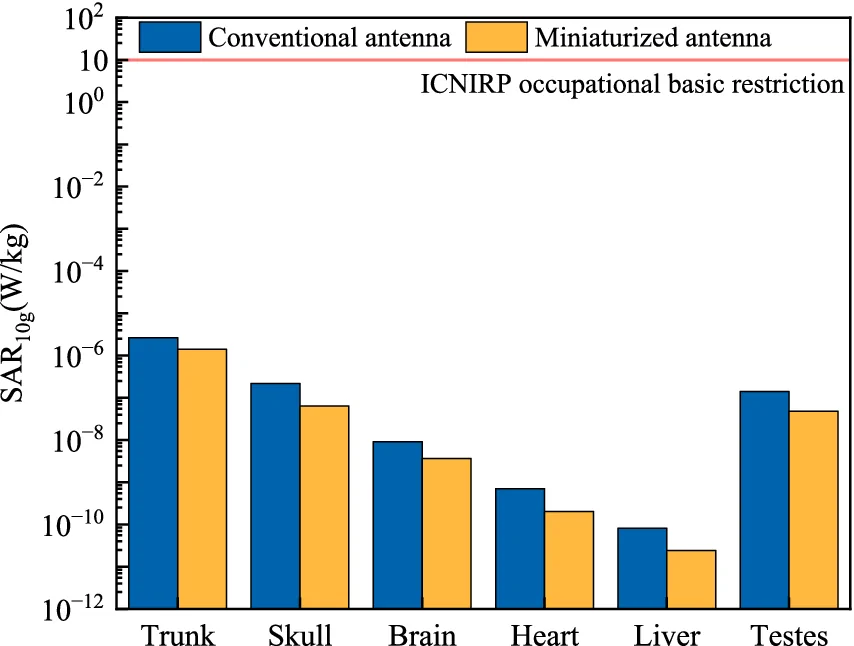
Comparison of SAR_10g_ values in different anatomical regions with the ICNIRP occupational basic restriction.

In comparison with the conventional track-side PIS antenna, the proposed track-side PIS antenna led to varying extents of reduction in SAR values across all tissues. When the antenna operates at 5.8 GHz, the SAR values in the trunk decreased by 81.75%, in the skull by 84.39%, in the brain by 85.56%, in the heart by 39.83%, in the liver by 63.30%, and in the testes by 86.33%. The peak spatial-average SAR_10g_ values of in the considered anatomical regions are compared with the applicable ICNIRP occupational basic restriction for localized SAR in the head and torso, which is 10 W/kg averaged over 10 g of contiguous tissue (25), as shown in Figure 12.

Figure 12 shows that the SAR_10g_ values for all examined anatomical regions are far below the ICNIRP occupational basic restriction of 10 W/kg. This indicates that the proposed track-side PIS antenna can effectively reduce SAR_10g_ values in various tissues, while maintaining compliance with the applicable ICNIRP occupational exposure limits.

### 3.5 Absolute temperature distribution in human body

To study the absolute temperature distribution in different human tissues within the metro tunnel, numerical simulations were performed using the transient Pennes bioheat equation:

$$\mathrm{pC}\frac{\partial T}{\partial t}=\nabla .(k\nabla T)+p_{b}C_{b}\omega_{b}(T_{b}-T)+Q_{\mathrm{met}}+Q_{\mathrm{ext}}$$ (4)

Where ρ is tissue density, C is tissue specific heat capacity, k is thermal conductivity, T is tissue temperature (°C), $T_{b}$ is blood temperature (°C), $\rho_{b}$ is blood density, $C_{b}$ is blood specific heat capacity, $\omega_{b}$ is blood perfusion rate, $Q_{\mathrm{met}}$ is the metabolic heat source, and $Q_{\mathrm{ext}}$ is the external heat source (absorbed RF power). The initial temperature was set to 36.6 °C. Adiabatic boundary conditions ( ∂T/∂n=0) were applied at all tissue-air interfaces. The external heat source $Q_{\mathrm{ext}}$ was derived voxel-wise from the computed SAR distribution as $Q_{\mathrm{ext}}=\rho \cdot \mathrm{SAR}$ .

**Figure 13:**
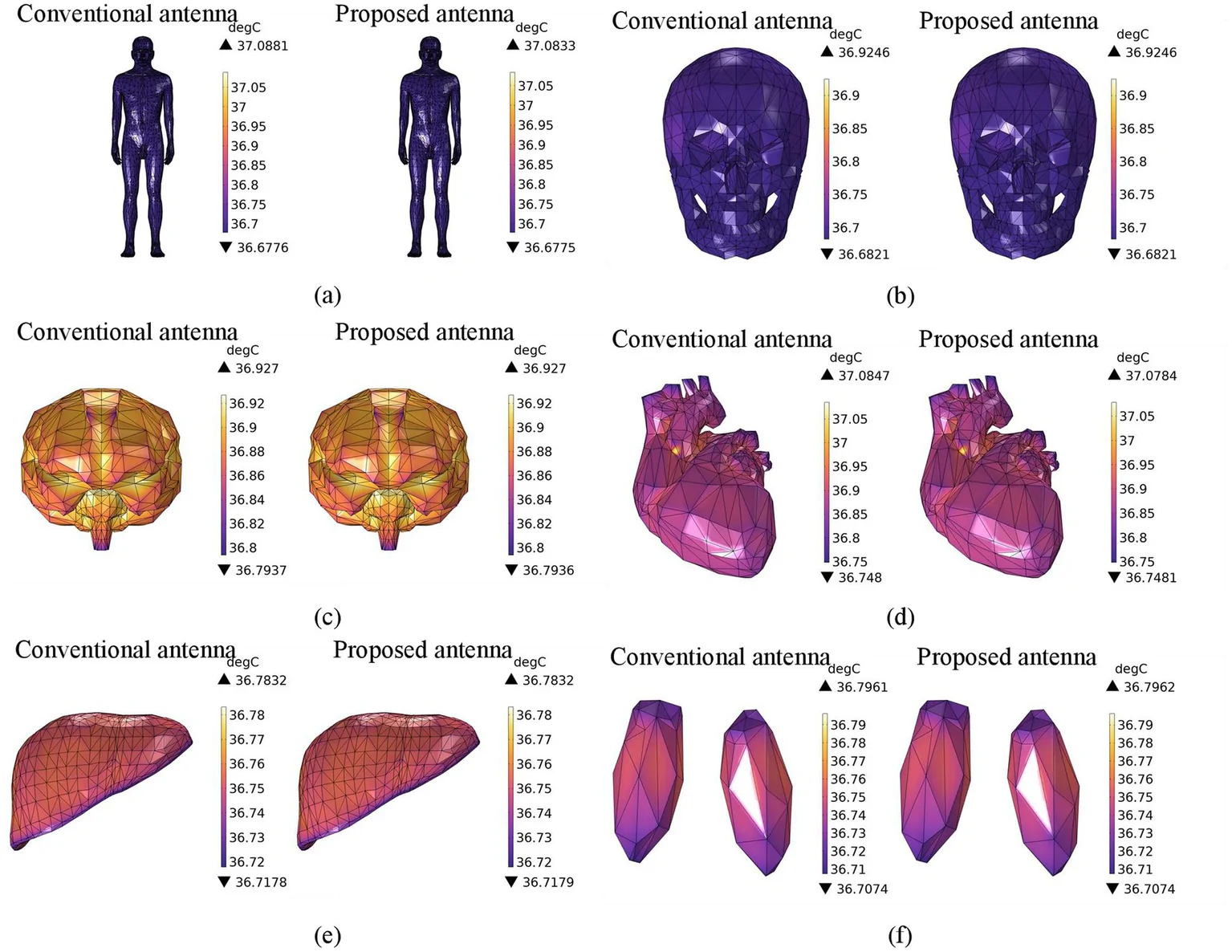
Comparison of absolute temperature distribution in different human tissues. **(a)** Trunk; **(b)** Skull; **(c)** Brain; **(d)** Heart; **(e)** Liver; **(f)** Testes.

To evaluate the transient thermal response of human tissues under the given electromagnetic irradiation condition, the simulation duration was set to 15 min. Figure 13 presents the absolute temperature distribution in different human tissues at the end of this simulation, under irradiation from conventional and proposed track-side PIS antennas.

From Figure 13, when the conventional track-side PIS antenna operates at 5.8 GHz, the temperature rises in the trunk, skull, brain, heart, liver, and testes are 0.4105 °C,0.2425 °C,0.1333 °C, 0.3367 °C, 0.0654 °C and 0.0887 °C, respectively. When the proposed track-side PIS antenna operates at 5.8 GHz, the corresponding temperature rises are 0.4058 °C, 0.2425 °C, 0.1334 °C, 0.3303 °C, 0.0653 °C and 0.0888 °C. According to the bioheat Equation 4, the temperature rise depends primarily on the biological tissue’s thermal parameters and the external heat source. Although the temperature rise varies across different tissues, the overall increase remains minimal due to the considerable distance between the radiation source and the human body. Consequently, the far-field effect of the track-side exposure source on human tissues is limited compared to the near-field effects of mobile terminals. Although the SAR values are substantially reduced, their absolute values remain very low under the present exposure conditions. Therefore, the RF-induced thermal effect is limited, and the large SAR reduction does not result in a comparable reduction in temperature rise.

## 4 Conclusion

This study focuses on the occupational electromagnetic exposure of track-side inspectors in metro tunnels and evaluates a compact metasurface track-side PIS antenna as a potential physical-layer exposure management approach. The principal findings are as follows: (1) The proposed track-side PIS antenna not only satisfies the communication requirements of the PIS dedicated network and the public 5G n79 band but also substantially enhances the electromagnetic environment within the tunnel. In comparison with the conventional track-side PIS antenna operating at 5.8 GHz, the proposed track-side PIS antenna reduces the peak field strength on the tunnel cross-section where the human body is located by 46.49%. (2) The proposed track-side PIS antenna notably suppresses the electromagnetic exposure levels in different tissues of track inspectors. Simulation results reveal that the peak local SAR values decline by 81.75% (trunk), 84.39% (skull), 85.56% (brain), 39.83% (heart), 63.30% (liver), and 86.33% (testes) and the peak electric field strength decreases by 57.11% (trunk), 60.00% (skull), 62.26% (brain), 24.08% (heart), 39.65% (liver), and 64.71% (testes). It demonstrates that the proposed track-side PIS antenna can effectively reduce electromagnetic energy absorption. In conclusion, this study integrates the occupational electromagnetic exposure safety assessment of track-side inspectors with a physical-layer antenna-based exposure mitigation approach for exploring occupational electromagnetic exposure reduction in metro tunnel environments.

## 5 Limitations

The exposure assessment was based on a standardized adult male model with a fixed posture and a simplified single-source scenario at 5.8 GHz, which may not fully represent practical metro tunnel environments involving different workers, postures, and multiple electromagnetic sources. In addition, uncertainties related to transmitter conditions, measurements, mesh resolution, and thermal modeling may influence the quantitative results.

## Data Availability

The original contributions presented in the study are included in the article/supplementary material, further inquiries can be directed to the corresponding author/s.
